# Quantitative risk assessment of UK food products cross-contaminated with allergens

**DOI:** 10.1186/2045-7022-5-S3-O10

**Published:** 2015-03-30

**Authors:** Benjamin C Remington, Joseph L Baumert, Marty W Blom, Geert F Houben, Steve L Taylor, Astrid G Kruizinga

**Affiliations:** 1 TNO, Zeist, The Netherlands; 2 FARRP, University of Nebraska, Lincoln, NE, USA

## 

Allergens in food pose a risk to allergic consumers, especially if they are present in food without declaration or warning. While there is EU regulation for allergens present as an ingredient, this is not the case for unintended allergen presence (UAP). Food companies use precautionary "may contain" labels to inform allergic individuals of a potential risk from UAPs. However, the use or absence of precautionary label has a limited correlation with the level of UAP and consequently the risk of an unexpected allergic reaction. Allergen risk assessment using probabilistic techniques enables estimation of the residual risk after the consumption of a product that unintendedly contains an allergen.

Previously, the UAP was determined among 500 packaged food products from 12 food groups in the UK, either containing a precautionary label or not (FSA,UK). In the present study, the UAP results were combined with food consumption data from the National Diet and Nutrition Survey (NDNS) in the UK and the allergen-specific population threshold distribution in a probabilistic model. Data used for the threshold distributions were gathered during the scientific review of the VITAL^®^ (Voluntary Incidental Trace Allergen Labelling). The allergens of interest were milk, wheat, peanut and hazelnut. The probabilistic model estimates the level of risk for objective allergic reactions posed to the allergic consumers in the UK and gives insight in the health implications of the measured unintended allergen levels.

Also, the levels of UAP were compared to a product-specific action level (ppm) based on the reference doses as determined for VITAL^®^ (mg protein) and the product specific consumption (kg).

The results of this study will assess the public health risks posed by the levels of allergen cross-contamination found to be present in food in the UK retail market and will provide insight regarding the implications of the potential implementation of the VITAL^®^ reference doses in evaluation of the use of advisory labeling in the UK.

